# Sodium penta­potassium penta­nickel tetra­(diphosphate), NaK_5_Ni_5_(P_2_O_7_)_4_

**DOI:** 10.1107/S160053681202017X

**Published:** 2012-05-12

**Authors:** Meryem Moutataouia, Mohammed Lamire, Mohamed Saadi, Lahcen El Ammari

**Affiliations:** a Laboratoire de Physico-Chimie des Matériaux Inorganiques, Faculté des Sciences Aïn Chock, Casablanca, Morocco; b Laboratoire de Chimie du Solide Appliquée, Faculté des Sciences, Université Mohammed V-Agdal, Avenue Ibn Batouta, BP 1014, Rabat, Morocco

## Abstract

The structure of the title compound, NaK_5_Ni_5_(P_2_O_7_)_4_, is characterized by the presence of two crystallographically independent P_2_O_7_ groups with different conformations. The conformation of the first P_2_O_7_ group is eclipsed, whereas that of the second is staggered. All atoms are in general positions except for two nickel and one potassium ions which lie on symmetry centers. Moreover, the structure exhibits disorder of the cationic sites with one general position fully occupied equally by Na^+^ and Ni^2+^ cations. This mixed site is surrounded by five O atoms forming a square-based pyramid. The crystal structure consists of edge-sharing [NiO_6_] octa­hedra forming infinite zigzag chains [Ni_3_O_14_] running parallel to [100]. Adjacent chains are connected through apices to P_2_O_7_ groups and to another [NiO_6_] or to a [KO_6_] octa­hedron. The resulting three-dimensional framework presents inter­secting tunnels running along the [010] and [001] directions in which the seven- and nine-coordinated potassium cations are located. The crystal structure of this new phosphate represents a new structural type.

## Related literature

For the crystal structure of nickel diphosphate, see: Lukaszewicz (1967 ▶); Pietraszko & Lukaszewicz (1968 ▶); Masse *et al.* (1979 ▶). For the structures of sodium and potassium nickel diphosphate, see: El Maadi *et al.* (1995 ▶); Erragh *et al.* (2000 ▶). For phosphates with mixed anions, see: Palkina & Maksimova (1980 ▶); Nagornyi *et al.* (1996 ▶); Sanz *et al.* (1999 ▶). For bond-valence analysis, see: Brown & Altermatt (1985 ▶).

## Experimental

### 

#### Crystal data

NaK_5_Ni_5_(P_2_O_7_)_4_
*M* *_r_* = 1207.80
Triclinic, 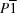

*a* = 7.188 (4) Å
*b* = 9.282 (5) Å
*c* = 10.026 (5) Å
α = 109.31 (2)°
β = 90.019 (12)°
γ = 104.066 (13)°
*V* = 610.0 (5) Å^3^
*Z* = 1
Mo *K*α radiation
μ = 5.31 mm^−1^
*T* = 296 K
0.20 × 0.14 × 0.11 mm

#### Data collection

Bruker APEXII CCD detector diffractometer
Absorption correction: multi-scan (*SADABS*; Sheldrick, 1999 ▶) *T*_min_ = 0.511, *T*_max_ = 0.638
13411 measured reflections
3608 independent reflections
3195 reflections with *I* > 2σ(*I*)
*R*_int_ = 0.033

#### Refinement

*R*[*F* ^2^ > 2σ(*F* ^2^)] = 0.027
*wR*(*F* ^2^) = 0.071
*S* = 1.03
3608 reflections
218 parameters
Δρ_max_ = 0.91 e Å^−3^
Δρ_min_ = −0.91 e Å^−3^

### 

Data collection: *APEX2* (Bruker, 2005 ▶); cell refinement: *SAINT* (Bruker, 2005 ▶); data reduction: *SAINT*; program(s) used to solve structure: *SHELXS97* (Sheldrick, 2008 ▶); program(s) used to refine structure: *SHELXL97* (Sheldrick, 2008 ▶); molecular graphics: *ORTEP-3 for Windows* (Farrugia, 1997 ▶) and *DIAMOND* (Brandenburg, 2006 ▶); software used to prepare material for publication: *PLATON* (Spek, 2009 ▶) and *publCIF* (Westrip, 2010 ▶).

## Supplementary Material

Crystal structure: contains datablock(s) I, global. DOI: 10.1107/S160053681202017X/br2200sup1.cif

Structure factors: contains datablock(s) I. DOI: 10.1107/S160053681202017X/br2200Isup2.hkl

Additional supplementary materials:  crystallographic information; 3D view; checkCIF report

## Figures and Tables

**Fig. 1.**
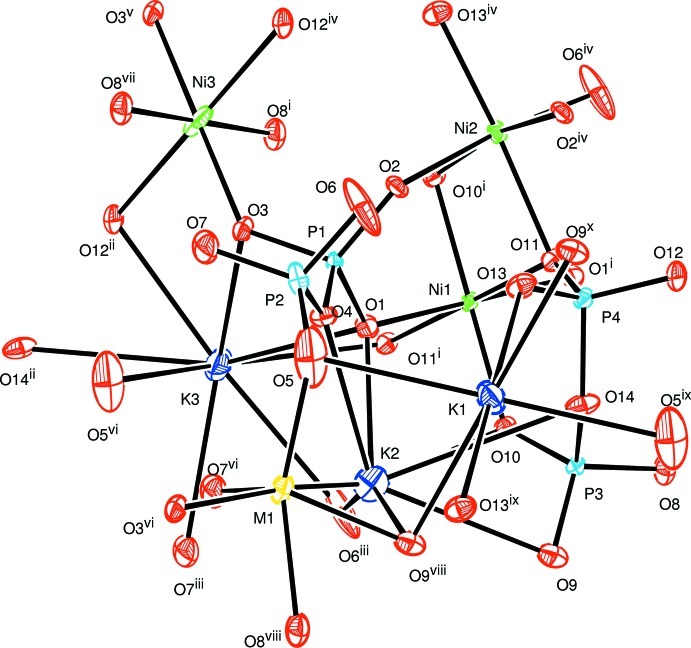
Plot of NaK5Ni5(P2O7)4 crystal structure showing polyhedra linkage. Displacement ellipsoids are drawn at the 50% probability level. Symmetry codes:(i) -x, -y, -z; (ii) -x + 1, -y, -z; (iii) -x + 1, -y + 1, -z; (iv) x + 1, y + 1, z; (v) x, y + 1, z; (vi) -x + 1, -y + 1, -z + 1; (vii) -x, -y, -z + 1; (viii) -x + 1, -y, -z + 1; (ix) x + 1, y, z; (x) x - 1, y, z; (xi) x - 1, y - 1, z; (xii) x, y - 1, z.

**Fig. 2.**
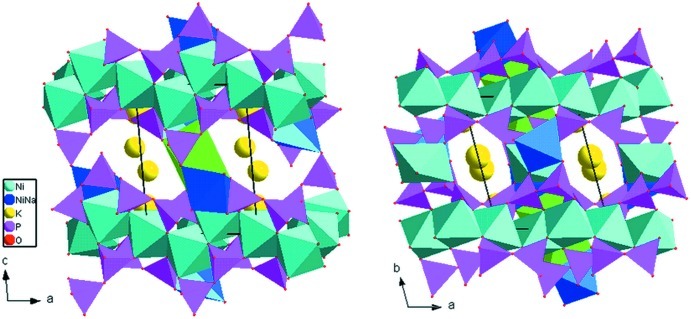
Projection views of the NaK5Ni5(P2O7)4 framework structure showing tunnels running along b and c directions where K2 and K3 atoms are located respectively.

## supplementary crystallographic information

### Comment

The most studied condensed phosphates are the diphosphates formerly called pyrophosphates. Most of these phosphates are polymorphic. The stability of each allotropic variety depends on several factors such as size and the electron affinity of the ions and synthesis conditions, in particular temperature and pressure. Thus in the case of Ni_2_P_2_O_7_, we note the existence of four allotropic varieties: the form α-Ni_2_P_2_O_7_ stable at room temperature and the γ-Ni_2_P_2_O_7_ determined by Lukaszewicz (1967), the high temperature form β-Ni_2_P_2_O_7_ obtained by Pietraszko & Lukaszewicz (1968) at 853 K and the δ-Ni_2_P_2_O_7_ discovered by Masse *et al.* (1979). The addition of an alkali cation like Na or K in this system leads to new compounds with different structures. Indeed we find K_2_NiP_2_O_7_ investigated by El Maadi *et al.* (1995), Na_7.39_Ni_4.24_(P_2_O_7_)_4_ by Erragh *et al.* 2000) and three compounds with mixed anions: K_2_Ni_4_(PO_4_)_2_(P_2_O_7_); Na_4_Ni_3_(PO_4_)_2_(P_2_O_7_) and Na_4_Ni_5_(PO_4_)_2_(P_2_O_7_)_2_ respectively, developed by Palkina & Maksimova (1980); Nagornyi *et al.* (1996) and Sanz *et al.* (1999) but to our knowledge no pyrophosphate with a mixture of two alkaline and nickel. The present work describes the synthesis method and the crystal structure of NaK_5_Ni_5_(P_2_O_7_)_4_ diphosphate from the X-ray diffraction data on single-crystal.

The partial three-dimensional plot in Fig.1 illustrates the connection ion-oxygen polyhedra in the crystal structure of the title compound. Among the 25 atoms include in the asymmetric unit of this structure, only three atoms (Ni1, Ni3 and K1) are in a special positions (1 symmetry). Furthermore, the structure is characterized by a cationic disorder in one general position fully occupied equally by Na1 and Ni4 atoms. This mixed site that will be noted M1 is surrounded by five oxygen atoms forming a square based pyramid. The conformations of the two crystallographically independent groups (P_2_O_7_) P1—O4—P2 and P3—O14—P4 are eclipsed and staggered, respectively as indicated by the torsion angles O1–P1–P2–O5 = 3.5 (2) ° and O8–P3–P4–O12 = -24.6 (2) °. The distances P1–P2 = 2.902 Å, P3–P4 = 3.015 Å and angles P1–O4–P2 = 127.65 (11)°, P3—O14—P4 = 135.07 (13)° are within the limits generally observed in condensed phosphate crystal chemistry. All three nickel atoms Ni1, Ni2 and Ni3 are surrounded by a roughly octahedral arrangement of six oxygen atoms except that in the mixed site (M1O_5_). The potassium atoms K1, K2 and K3 are coordinated to six, seven and nine oxygen atoms, respectively.

The structure of NaK_5_Ni_5_(P_2_O_7_)_4_ consists of edge-sharing [Ni1O_6_] and [Ni2O_6_] octahedra forming an infinite zigzag chains [Ni_3_O_14_] running parallel to [100], as shown in Fig. 2. Adjacent chains are connected by P_2_O_7_ groups, M1O_5_ square-pyramid and [Ni3O_6_] or [K1O_6_] octahedra *via* vertices in the way to build three-dimensional network.

The resulting 3-D framework presents intersecting tunnels running along the [010] and [001] directions, where the K2 and K3 potassium cations are located (Fig.2). Probably, the structure of this phosphate represents a new structural type.

### Experimental

NaK_5_Ni_5_(P_2_O_7_)_4_ is obtained during the preparation of a mixture of triphosphate. Indeed, the powder of "NaK_2_NiP_3_O_10_" synthesized by wet process is introduced into a platinum crucible, and then gradually heated to a temperature above its melting point (1073 K) for 2 h, followed by slow cooling of the order of 10 °K per hour up to 673 °K. Then the furnace is shuts down and the cooling is continued until room temperature. Small colourless single crystals were isolated from the mixture of phases. The study of crystal structure by X-ray diffraction leads to the formula of the new phosphate: NaK_5_Ni_5_(P_2_O_7_)_4_.

### Refinement

The atomic displacement factors of O5 and O6 atoms are very large compared to other oxygen atoms because the O5 is linked to Ni4 and Na1 occupying the disordered site and O6 is related to K2 and K3 that are in the tunnels. This explains the large displacement of these atoms. The highest peak and the deepest hole in the final Fourier map are at 0.76 Å and 0.49 Å, respectively, from Na1 and K2. The refinement of the occupancy rates of the mixed site of Na1 and Ni4 led to 0.5 for Na and 0.5 for Ni. These values are in good agreement with the charge balance and the bond valence analysis which gives occupation numbers of 0.60 for Ni and 0.40 for Na (Brown & Altermatt, 1985). The not significant bonds and angles were removed from the CIF file.

### Crystal data

**Table:**

NaK_5_Ni_5_(P_2_O_7_)_4_	*Z* = 1
*M**_r_* = 1207.80	*F*(000) = 590
Triclinic, *P*1	*D*_x_ = 3.288 Mg m^−^^3^
Hall symbol: -p 1	Mo *K*α radiation, λ = 0.71073 Å
*a* = 7.188 (4) Å	Cell parameters from 3608 reflections
*b* = 9.282 (5) Å	θ = 2.6–30.2°
*c* = 10.026 (5) Å	µ = 5.31 mm^−^^1^
α = 109.31 (2)°	*T* = 296 K
β = 90.019 (12)°	Block, colourless
γ = 104.066 (13)°	0.20 × 0.14 × 0.11 mm
*V* = 610.0 (5) Å^3^	

### Data collection

**Table:**

Bruker APEXII CCD detector diffractometer	3608 independent reflections
Radiation source: fine-focus sealed tube	3195 reflections with *I* > 2σ(*I*)
Graphite monochromator	*R*_int_ = 0.033
ω and φ scans	θ_max_ = 30.2°, θ_min_ = 2.6°
Absorption correction: multi-scan (*SADABS*; Sheldrick, 1999)	*h* = −10→10
*T*_min_ = 0.511, *T*_max_ = 0.638	*k* = −13→13
13411 measured reflections	*l* = −14→14

### Refinement

**Table:**

Refinement on *F*^2^	Primary atom site location: structure-invariant direct methods
Least-squares matrix: full	Secondary atom site location: difference Fourier map
*R*[*F*^2^ > 2σ(*F*^2^)] = 0.027	*w* = 1/[σ^2^(*F*_o_^2^) + (0.034*P*)^2^ + 1.127*P*] where *P* = (*F*_o_^2^ + 2*F*_c_^2^)/3
*wR*(*F*^2^) = 0.071	(Δ/σ)_max_ < 0.001
*S* = 1.03	Δρ_max_ = 0.91 e Å^−^^3^
3608 reflections	Δρ_min_ = −0.91 e Å^−^^3^
218 parameters	Extinction correction: *SHELXL97* (Sheldrick, 2008), Fc^*^=kFc[1+0.001xFc^2^λ^3^/sin(2θ)]^-1/4^
0 restraints	Extinction coefficient: 0.0078 (9)

### Special details

**Table:**

Geometry. All s.u.'s (except the s.u. in the dihedral angle between two l.s. planes) are estimated using the full covariance matrix. The cell s.u.'s are taken into account individually in the estimation of s.u.'s in distances, angles and torsion angles; correlations between s.u.'s in cell parameters are only used when they are defined by crystal symmetry. An approximate (isotropic) treatment of cell s.u.'s is used for estimating s.u.'s involving l.s. planes.
Refinement. Refinement of *F*^2^ against all reflections. The weighted *R*-factor *wR* and goodness of fit *S* are based on *F*^2^, conventional *R*-factors *R* are based on *F*, with *F* set to zero for negative *F*^2^. The threshold expression of *F*^2^ > 2σ(*F*^2^) is used only for calculating *R*-factors(gt) *etc*. and is not relevant to the choice of reflections for refinement. *R*-factors based on *F*^2^ are statistically about twice as large as those based on *F*, and *R*- factors based on all data will be even larger.

### Fractional atomic coordinates and isotropic or equivalent isotropic displacement parameters (Å^2^)

**Table:**

	*x*	*y*	*z*	*U*_iso_*/*U*_eq_	Occ. (<1)
Ni1	0.0000	0.0000	0.0000	0.00836 (10)	
Ni2	0.36001 (4)	−0.04138 (3)	−0.12590 (3)	0.01024 (8)	
Ni3	0.5000	0.5000	0.0000	0.02272 (13)	
Ni4	0.42559 (7)	0.37578 (6)	0.68239 (5)	0.01395 (11)	0.50
Na1	0.42559 (7)	0.37578 (6)	0.68239 (5)	0.01395 (11)	0.50
K1	0.5000	0.0000	0.5000	0.02299 (18)	
K2	0.05087 (10)	0.19327 (8)	0.42045 (7)	0.02536 (14)	
K3	0.00287 (10)	0.45372 (7)	0.18072 (8)	0.02541 (14)	
P1	0.36193 (8)	0.26003 (7)	0.17071 (6)	0.00759 (12)	
P2	0.65917 (10)	0.34427 (8)	0.39912 (7)	0.01475 (14)	
P3	−0.14668 (8)	−0.18481 (7)	0.22059 (6)	0.00879 (12)	
P4	0.25079 (8)	−0.18870 (7)	0.12603 (7)	0.00936 (12)	
O1	0.1486 (2)	0.1946 (2)	0.16422 (19)	0.0129 (3)	
O2	0.4605 (2)	0.1478 (2)	0.06750 (18)	0.0110 (3)	
O3	0.4162 (3)	0.4235 (2)	0.15797 (19)	0.0134 (3)	
O4	0.4368 (2)	0.2883 (2)	0.33014 (19)	0.0118 (3)	
O5	0.6436 (5)	0.3267 (3)	0.5429 (3)	0.0456 (8)	
O6	0.7578 (3)	0.2302 (3)	0.3004 (3)	0.0410 (7)	
O7	0.7412 (3)	0.5116 (2)	0.4030 (2)	0.0239 (4)	
O8	−0.2634 (3)	−0.3502 (2)	0.1363 (2)	0.0172 (4)	
O9	−0.2049 (3)	−0.1394 (2)	0.3710 (2)	0.0174 (4)	
O10	−0.1531 (2)	−0.0619 (2)	0.15276 (19)	0.0120 (3)	
O11	0.1762 (2)	−0.1410 (2)	0.00776 (19)	0.0107 (3)	
O12	0.2715 (3)	−0.3547 (2)	0.0742 (2)	0.0167 (4)	
O13	0.4217 (3)	−0.0687 (2)	0.21951 (19)	0.0143 (3)	
O14	0.0753 (3)	−0.1883 (2)	0.2327 (2)	0.0164 (4)	

### Atomic displacement parameters (Å^2^)

**Table:**

	*U*^11^	*U*^22^	*U*^33^	*U*^12^	*U*^13^	*U*^23^
Ni1	0.00652 (18)	0.00716 (18)	0.0118 (2)	0.00142 (14)	0.00205 (15)	0.00396 (15)
Ni2	0.00772 (14)	0.00786 (14)	0.01396 (16)	0.00273 (11)	0.00143 (11)	0.00166 (11)
Ni3	0.0308 (3)	0.0183 (2)	0.0120 (2)	−0.0114 (2)	−0.0028 (2)	0.00858 (19)
Ni4	0.0144 (2)	0.0095 (2)	0.0161 (2)	−0.00049 (17)	−0.00043 (18)	0.00461 (18)
Na1	0.0144 (2)	0.0095 (2)	0.0161 (2)	−0.00049 (17)	−0.00043 (18)	0.00461 (18)
K1	0.0363 (5)	0.0181 (4)	0.0159 (4)	0.0108 (3)	0.0048 (3)	0.0049 (3)
K2	0.0255 (3)	0.0285 (3)	0.0226 (3)	0.0048 (3)	−0.0035 (2)	0.0110 (3)
K3	0.0256 (3)	0.0181 (3)	0.0365 (4)	0.0065 (2)	0.0080 (3)	0.0139 (3)
P1	0.0070 (2)	0.0060 (2)	0.0097 (3)	0.00174 (19)	0.0021 (2)	0.0025 (2)
P2	0.0162 (3)	0.0093 (3)	0.0164 (3)	0.0049 (2)	−0.0048 (2)	0.0001 (2)
P3	0.0081 (3)	0.0080 (3)	0.0102 (3)	0.0015 (2)	0.0023 (2)	0.0034 (2)
P4	0.0078 (3)	0.0087 (3)	0.0124 (3)	0.0021 (2)	0.0017 (2)	0.0047 (2)
O1	0.0082 (8)	0.0119 (8)	0.0164 (8)	0.0017 (6)	0.0021 (6)	0.0025 (7)
O2	0.0098 (8)	0.0098 (7)	0.0116 (8)	0.0037 (6)	0.0029 (6)	0.0004 (6)
O3	0.0178 (9)	0.0092 (8)	0.0141 (8)	0.0024 (7)	0.0027 (7)	0.0058 (7)
O4	0.0097 (8)	0.0142 (8)	0.0111 (8)	0.0020 (6)	0.0019 (6)	0.0049 (7)
O5	0.088 (2)	0.0238 (12)	0.0236 (12)	0.0141 (13)	−0.0258 (13)	0.0071 (10)
O6	0.0198 (11)	0.0277 (12)	0.0533 (16)	0.0167 (10)	−0.0176 (11)	−0.0223 (11)
O7	0.0176 (9)	0.0138 (9)	0.0312 (11)	−0.0018 (7)	0.0042 (8)	−0.0001 (8)
O8	0.0208 (9)	0.0105 (8)	0.0165 (9)	0.0004 (7)	−0.0026 (7)	0.0024 (7)
O9	0.0195 (9)	0.0202 (9)	0.0126 (8)	0.0070 (7)	0.0047 (7)	0.0043 (7)
O10	0.0106 (8)	0.0115 (8)	0.0174 (8)	0.0050 (6)	0.0044 (6)	0.0081 (7)
O11	0.0085 (7)	0.0118 (8)	0.0140 (8)	0.0033 (6)	0.0013 (6)	0.0066 (6)
O12	0.0211 (9)	0.0093 (8)	0.0218 (9)	0.0061 (7)	0.0042 (7)	0.0065 (7)
O13	0.0108 (8)	0.0177 (9)	0.0135 (8)	0.0005 (7)	0.0001 (6)	0.0062 (7)
O14	0.0102 (8)	0.0264 (10)	0.0195 (9)	0.0071 (7)	0.0051 (7)	0.0150 (8)

### Geometric parameters (Å, º)

**Table:**

Ni1—O10^i^	2.0403 (19)	K3—O6^x^	2.953 (3)
Ni1—O10	2.0403 (19)	K3—O12^i^	2.971 (3)
Ni1—O11	2.0487 (18)	K3—O3	3.053 (3)
Ni1—O11^i^	2.0487 (18)	K3—O8^v^	3.066 (2)
Ni1—O1	2.052 (2)	K3—O14^v^	3.098 (3)
Ni1—O1^i^	2.052 (2)	P1—O1	1.4986 (19)
Ni1—Ni2	2.9329 (14)	P1—O2	1.5178 (18)
Ni2—O2^ii^	1.9963 (19)	P1—O3	1.5200 (19)
Ni2—O10^i^	2.0171 (19)	P1—O4	1.602 (2)
Ni2—O6^ii^	2.024 (2)	P2—O5	1.505 (3)
Ni2—O13^ii^	2.0595 (19)	P2—O6	1.509 (2)
Ni2—O2	2.120 (2)	P2—O7	1.509 (2)
Ni2—O11	2.1504 (18)	P2—O4	1.631 (2)
Ni2—Ni2^ii^	2.9960 (14)	P3—O8	1.514 (2)
Ni3—O3	1.9781 (19)	P3—O9	1.515 (2)
Ni3—O3^iii^	1.9781 (19)	P3—O10	1.5178 (18)
Ni3—O8^i^	2.070 (2)	P3—O14	1.609 (2)
Ni3—O8^iv^	2.070 (2)	P4—O12	1.500 (2)
Ni3—O12^ii^	2.342 (2)	P4—O13	1.508 (2)
Ni3—O12^v^	2.342 (2)	P4—O11	1.5333 (19)
M1—O3^vi^	2.079 (2)	P4—O14	1.654 (2)
M1—O7^vi^	2.112 (2)	O2—Ni2^ii^	1.9963 (19)
M1—O5	2.132 (4)	O3—M1^vi^	2.079 (2)
M1—O8^vii^	2.208 (2)	O6—Ni2^ii^	2.024 (2)
M1—O9^vii^	2.269 (2)	O6—K2^ix^	2.574 (3)
K1—O13^viii^	2.696 (2)	O6—K3^ix^	2.953 (3)
K1—O13	2.696 (2)	O7—M1^vi^	2.112 (2)
K1—O5^viii^	2.839 (3)	O7—K2^vi^	2.770 (2)
K1—O5	2.839 (3)	O7—K3^ix^	2.915 (2)
K1—O9^ix^	2.842 (2)	O8—Ni3^xi^	2.070 (2)
K1—O9^vii^	2.842 (2)	O8—M1^vii^	2.208 (2)
K2—O6^x^	2.574 (3)	O8—K3^xii^	3.066 (2)
K2—O9^vii^	2.609 (2)	O9—M1^vii^	2.269 (2)
K2—O1	2.667 (2)	O9—K2^vii^	2.609 (2)
K2—O7^vi^	2.770 (2)	O9—K1^x^	2.842 (2)
K2—O4	2.938 (2)	O10—Ni2^i^	2.0171 (19)
K2—O10	3.011 (2)	O11—K3^i^	2.865 (2)
K2—O9	3.064 (3)	O12—Ni3^xii^	2.342 (2)
K3—O12^v^	2.760 (2)	O12—K3^xii^	2.760 (2)
K3—O1	2.805 (2)	O12—K3^i^	2.971 (2)
K3—O11^i^	2.865 (2)	O13—Ni2^ii^	2.0595 (19)
K3—O7^x^	2.915 (3)	O14—K3^xii^	3.098 (3)
			
O10^i^—Ni1—O10	180.00 (14)	O9^vii^—K2—O4	89.43 (7)
O10^i^—Ni1—O11	89.76 (7)	O1—K2—O4	51.46 (5)
O10—Ni1—O11	90.24 (7)	O7^vi^—K2—O4	67.65 (6)
O10^i^—Ni1—O11^i^	90.24 (7)	O6^x^—K2—O10	60.85 (7)
O10—Ni1—O11^i^	89.76 (7)	O9^vii^—K2—O10	124.00 (7)
O11—Ni1—O11^i^	180.00 (7)	O1—K2—O10	58.11 (6)
O10^i^—Ni1—O1	94.64 (8)	O7^vi^—K2—O10	158.76 (6)
O10—Ni1—O1	85.36 (8)	O4—K2—O10	101.33 (6)
O11—Ni1—O1	95.94 (8)	O6^x^—K2—O9	84.11 (8)
O11^i^—Ni1—O1	84.06 (8)	O9^vii^—K2—O9	81.83 (7)
O10^i^—Ni1—O1^i^	85.36 (8)	O1—K2—O9	105.24 (6)
O10—Ni1—O1^i^	94.64 (8)	O7^vi^—K2—O9	151.80 (7)
O11—Ni1—O1^i^	84.06 (8)	O4—K2—O9	129.11 (6)
O11^i^—Ni1—O1^i^	95.94 (8)	O10—K2—O9	49.03 (5)
O1—Ni1—O1^i^	180.00 (15)	O12^v^—K3—O1	106.14 (7)
O2^ii^—Ni2—O10^i^	169.20 (7)	O12^v^—K3—O11^i^	117.27 (7)
O2^ii^—Ni2—O6^ii^	93.83 (9)	O1—K3—O11^i^	57.90 (6)
O10^i^—Ni2—O6^ii^	89.84 (9)	O12^v^—K3—O7^x^	134.29 (7)
O2^ii^—Ni2—O13^ii^	89.76 (8)	O1—K3—O7^x^	112.03 (6)
O10^i^—Ni2—O13^ii^	99.83 (8)	O11^i^—K3—O7^x^	104.10 (6)
O6^ii^—Ni2—O13^ii^	97.60 (11)	O12^v^—K3—O6^x^	172.29 (6)
O2^ii^—Ni2—O2	86.65 (8)	O1—K3—O6^x^	66.64 (6)
O10^i^—Ni2—O2	88.70 (8)	O11^i^—K3—O6^x^	61.89 (7)
O6^ii^—Ni2—O2	174.32 (10)	O7^x^—K3—O6^x^	50.52 (6)
O13^ii^—Ni2—O2	88.07 (8)	O12^v^—K3—O12^i^	91.74 (7)
O2^ii^—Ni2—O11	82.20 (7)	O1—K3—O12^i^	106.98 (6)
O10^i^—Ni2—O11	87.57 (7)	O11^i^—K3—O12^i^	51.26 (5)
O6^ii^—Ni2—O11	91.53 (11)	O7^x^—K3—O12^i^	100.05 (8)
O13^ii^—Ni2—O11	168.23 (7)	O6^x^—K3—O12^i^	93.03 (7)
O2—Ni2—O11	82.93 (8)	O12^v^—K3—O3	58.80 (6)
O3—Ni3—O3^iii^	180.00 (9)	O1—K3—O3	50.60 (5)
O3—Ni3—O8^i^	93.37 (8)	O11^i^—K3—O3	96.32 (6)
O3^iii^—Ni3—O8^i^	86.63 (8)	O7^x^—K3—O3	137.10 (7)
O3—Ni3—O8^iv^	86.63 (8)	O6^x^—K3—O3	113.49 (6)
O3^iii^—Ni3—O8^iv^	93.37 (8)	O12^i^—K3—O3	121.90 (6)
O8^i^—Ni3—O8^iv^	180.00 (17)	O12^v^—K3—O8^v^	83.94 (7)
O3—Ni3—O12^ii^	97.37 (7)	O1—K3—O8^v^	161.23 (6)
O3^iii^—Ni3—O12^ii^	82.63 (7)	O11^i^—K3—O8^v^	103.49 (6)
O8^i^—Ni3—O12^ii^	100.17 (8)	O7^x^—K3—O8^v^	67.60 (7)
O8^iv^—Ni3—O12^ii^	79.83 (8)	O6^x^—K3—O8^v^	103.75 (7)
O3—Ni3—O12^v^	82.63 (7)	O12^i^—K3—O8^v^	56.06 (6)
O3^iii^—Ni3—O12^v^	97.37 (7)	O3—K3—O8^v^	142.67 (6)
O8^i^—Ni3—O12^v^	79.83 (8)	O12^v^—K3—O14^v^	50.49 (6)
O8^iv^—Ni3—O12^v^	100.17 (8)	O1—K3—O14^v^	149.36 (6)
O12^ii^—Ni3—O12^v^	180.0	O11^i^—K3—O14^v^	145.51 (6)
O3^vi^—M1—O7^vi^	97.28 (9)	O7^x^—K3—O14^v^	84.43 (6)
O3^vi^—M1—O5	100.21 (10)	O6^x^—K3—O14^v^	134.95 (6)
O7^vi^—M1—O5	107.23 (9)	O12^i^—K3—O14^v^	94.58 (6)
O3^vi^—M1—O8^vii^	80.71 (8)	O3—K3—O14^v^	99.55 (5)
O7^vi^—M1—O8^vii^	100.81 (9)	O8^v^—K3—O14^v^	48.38 (6)
O5—M1—O8^vii^	151.53 (8)	O1—P1—O2	113.34 (11)
O3^vi^—M1—O9^vii^	146.47 (7)	O1—P1—O3	112.74 (10)
O7^vi^—M1—O9^vii^	97.27 (9)	O2—P1—O3	111.86 (11)
O5—M1—O9^vii^	104.03 (9)	O1—P1—O4	104.08 (10)
O8^vii^—M1—O9^vii^	66.96 (7)	O2—P1—O4	109.57 (10)
O13^viii^—K1—O13	180.0	O3—P1—O4	104.53 (10)
O13^viii^—K1—O5^viii^	91.78 (7)	O5—P2—O6	112.93 (17)
O13—K1—O5^viii^	88.22 (7)	O5—P2—O7	114.35 (14)
O13^viii^—K1—O5	88.22 (7)	O6—P2—O7	112.17 (15)
O13—K1—O5	91.78 (7)	O5—P2—O4	104.13 (15)
O5^viii^—K1—O5	180.00 (14)	O6—P2—O4	104.89 (12)
O13^viii^—K1—O9^ix^	104.71 (6)	O7—P2—O4	107.41 (11)
O13—K1—O9^ix^	75.29 (6)	O8—P3—O9	109.31 (12)
O5^viii^—K1—O9^ix^	75.29 (9)	O8—P3—O10	114.40 (11)
O5—K1—O9^ix^	104.71 (9)	O9—P3—O10	112.48 (11)
O13^viii^—K1—O9^vii^	75.29 (6)	O8—P3—O14	107.93 (11)
O13—K1—O9^vii^	104.71 (6)	O9—P3—O14	105.88 (11)
O5^viii^—K1—O9^vii^	104.71 (9)	O10—P3—O14	106.35 (10)
O5—K1—O9^vii^	75.29 (9)	O12—P4—O13	114.34 (12)
O9^ix^—K1—O9^vii^	180.0	O12—P4—O11	112.79 (11)
O6^x^—K2—O9^vii^	149.78 (9)	O13—P4—O11	114.65 (11)
O6^x^—K2—O1	74.28 (9)	O12—P4—O14	105.83 (11)
O9^vii^—K2—O1	135.37 (7)	O13—P4—O14	103.88 (11)
O6^x^—K2—O7^vi^	107.73 (8)	O11—P4—O14	103.90 (10)
O9^vii^—K2—O7^vi^	75.36 (8)	P1—O4—P2	127.65 (11)
O1—K2—O7^vi^	102.63 (7)	P3—O14—P4	135.07 (13)
O6^x^—K2—O4	119.96 (9)		
			
O1—P1—P2—O5	3.52 (17)	O8—P3—P4—O12	−24.60 (12)

Symmetry codes: (i) −*x*, −*y*, −*z*; (ii) −*x*+1, −*y*, −*z*; (iii) −*x*+1, −*y*+1, −*z*; (iv) *x*+1, *y*+1, *z*; (v) *x*, *y*+1, *z*; (vi) −*x*+1, −*y*+1, −*z*+1; (vii) −*x*, −*y*, −*z*+1; (viii) −*x*+1, −*y*, −*z*+1; (ix) *x*+1, *y*, *z*; (x) *x*−1, *y*, *z*; (xi) *x*−1, *y*−1, *z*; (xii) *x*, *y*−1, *z*.
